# Dental Anatomy
*Read at the Alumni Meeting of the University of Tennessee, Dental Department, January, 1898.


**Published:** 1898-07

**Authors:** A. H. Thompson

**Affiliations:** Topeka, Kas.


AMERICAN JOURNAL

—OF—

DENTAL SCIENCE.

VOL. XXXII. BALTIMORE, JULY, 1898. NO. 3.

### Selections.

ARTICLE I.

### DENTAL ANATOMY.*

DR. A. H. THOMPSON, TOPEKA, KAS.

I was somewhat surprised, on looking over the programme of this meeting yesterday afternoon, to find myself booked for a " lecture " before the Alumni Association, without even having a subject assigned to me. On remonstrating with the chairman, he replied that they would graciously allow me to select my own subject, which, I was assured, was a very great privilege.

Now, it so happens that there is only one subject with which I am sufficiently familiar—with which my intellectual battery is charged, so to speak—upon which I could risk an impromptu lecture or address. It is the one subject for which I have neglected all others of late years, and that is " Dental Anatomy," which must be my excuse for its selection. And in speaking upon this subject I will

*Read at the Alumni Meeting of the University of Tennessee, Dental Department, January, 1898.

need to first ask the pardon and indulgence of the older members of my profession present, for I choose to address myself mainly to the students present, and must therefore be elementary in the presentation of the main principles involved.

*Dental Anatomy—i. e.*, the macroscopic or external anatomy of the teeth, as distinguished from the microscopic or internal anatomy, the histology of the dental tissues. This study, strange as it may seem, has not received the special attention in the curriculum of our education that its importance certainly warrants. When we consider that the most of working hours in our daily practice are taken up in dealing with and the restoration of the external forms of the teeth in order to the preservation of their practical and functional effectiveness and esthetic offices, surely nothing can be more important than a thorough knowledge of tooth forms as regards their anatomical, mechanical and esthetic elements. And if this knowledge is important, it follows that it is also important that we should know the path by which the teeth have attained these forms through a study of the teeth of lower animals, comparative dental anatomy or odontography. By their study we learn how tooth-forms are acquired both in man and animals by the process of evolution, and also of the philosophy of tooth-forms in their adaptation to their various functional purposes. If we are to understand the anatomy of the human teeth and the functions they are to perform, surely no study can be more important as having a greater bearing upon our everyday work in serving our fellowmen.

In the time at our disposal it will be possible only to speak briefly of the general principles involved in this study, and not to go into details, interesting as that might be in some directions. One of the first and most important of these underlying principles is that of dental occlusion, and the force and variety of jaw-movements. The teeth, as you well understand, are merely the armament of the masticating apparatus. They are but modified dermal appendages placed upon the margins of the jaws to bear the impact of the work of food-reduction preparatory to its digestion. They receive the masticating force, the jaws and muscles do the work. Therefore, the force of jaw movement comes upon them with tremendous effect, the importance of which cannot be over-estimated. Occlusion is the force that dictates tooth-forms and modifies the jaws as well. For the endurance of this force in all its variety the teeth were erected, and by it they are maintained in perfection of form or are modified to adapt them to varying purposes by the variety of food presented. When this force is lessened the teeth are reduced or altered by disuse in response to the law of economy of growth, like all other organs which owe their existence to use and their atrophy and abortion to disuse. In this connection it is well to remind you of the importance of the force of occlusion to us in a practical way and the necessity of appreciating its tremendous power, even in man. It has such a bearing upon all our operations upon the natural teeth, as well as in prosthesis, that we should, to get a better understanding of its power and influence, study it in a philosophical way among lower animals as well. We observe among the latter two principal kinds of jaw-movement—*i. e.*, the vertical closure of the mandible, as observed in the *Carnivora*, and the lateral or horizontal movement, as in *Herbivora*. These two great classes present the two extremes, but between these there are all varieties of graded forms of movement, and consequently of tooth-forms and jaw-mechanism. We notice also the effects that the extreme movements have upon the teeth as well as the jaws, for both are modified together. Thus in the carnivora the jaws possess but one movement, that of opening and closing, which are adapted to the mere cutting of flesh. The jaw articulation is a mere hinge, which allows of but the one movement. To correspond to this movement the teeth are elongated vertically into long points and blades for the cutting and dividing of flesh—*i. e.*, they are developed in the direction of greatest strain, vertically. That is the direction of use. On the contrary, the herbivorous animals have extensive lateral and horizontal movement of the jaws, so that the articulation is open and loose to allow of the lateral movement of the jaw. The teeth are also developed laterally to correspond to this movement, in response to lateral strain, as opposed to the teeth of the carnivora, which are developed vertically in response to vertical strain. The lateral movement has been developed by the necessity of the extensive grinding and manipulation which the food of the herbivora requires. Between these extremes are various grades of organization as the food of animals varies and becomes more omnivorous. In this class man belongs, for his teeth are neither carnivorous nor herbivorous, but are intermediate, as his food is mixed and intermediate.

Another great principle is that of the *evolution of tooth-forms.* We observe that the simple mechanical element of the tooth is that of the single cone, which is the primitive type of tooth, from which all later and subsequent forms were evolved by modification and duplication. The simple cone is the form presented by the lowest classes of vertebrates, the fishes and reptiles, in which the function of the teeth is mere prehension—the seizing and holding of prey preparatory to its deglutition, but without any preparatory mastication. In the lowest mammals the cone is modified into the simple molar for crushing, as in the armadillos and sloths. In some others, as the dolphin, it is still the conical form of the reptiles. In the lizards tubercles begin to be added to form tuberculate teeth, by duplication of the cones. In higher mammals the cones are modified or duplicated to form two or three or more tubercles. Thus the incisor of man is a single cone with the base flattened to an edge for cutting. The canine is a single cone with the base compressed to a tribedral point. The biscuspids are composed of two cones fused together, which are plainly marked in the upper bicuspids. The upper molars are composed of three cones and a fourth added tubercle. The lower molars of man are composed of four cones. In other mammalia the primitive cone is modified or duplicated in various directions, in obedience to the force of occlusion, which depends in its turn upon food selection. The first, the primary cone, is called the protocone, as of the single-coned teeth of reptiles. To this was added next the inner cone of the upper bicuspids—called the detercone. The upper molars were the first formed of three cones in a row —the protocone, the metacone and the paracone, which formed the triconodont form. The second and third cones were then moved to the buccal side and formed the trigon of the three cones of the upper molar. The lower molar was also formed of three cones in triangular arrangement, which passed the upper trigon with a shearing motion. This primitive form of crown is still preserved in some animals, as in the American opossum and others. Then the heel or talon was thrown out, which carries the fourth tubercle above and the two extra tubercles below, the hypocone and hypoconid. These strike in the center of the opposing trigons and form the grinding apparatus of the molars.

The next great principle which we will have time to notice is that of the *functional forms of the teeth.* In the highest mammals these forms are various as employed for various purposes. First, the function of prehension is performed by the canines, which are sharp-pointed for seizing and tearing, especially in the carnivora; second, the cutting or dividing is performed by the incisors as in man and the herbivora, but by the long-bladed sectorial teeth of the carnivora; third, the crushing function is performed in the higher mammals by the biscuspids or premolars; and fourth, the masticating and insalivating functions are performed by the broad triturating molars. These various functions are gradually developed as the scale of animal life is followed from the lowest to the highest forms, and afford beautiful examples of the adaptation of tools to material in response to the various kinds of work they are called upon to perform.

In replying to question: Deciduous teeth occur in nearly all of the highest mammals, the premolars, canines and incisors being lost from the first set and are replaced by a second or succedaneous set. The true molars, as in man, are produced but once. Mammals are divided into two great classes with reference to the presence or absence of two sets of teeth. Those having but one set of teeth which persist through life are called monophyodonts, while those having two sets are called diphyodonts. Some of the higher mammals, as the common rat, have but one set, which persist through life.

In answer to question, some of the practical applications of these studies might be enumerated as follows: First, the study as an intellectual pursuit. Everyone should have a "hobby," something in which he excels and that he can follow into its higher branches. Such a study, or any study that involves intellectual work, not only conduces to strengthening the mind and gives intellectual pleasure, but it is really a safeguard against moral dangers. The man who is interested in an intellectual subject and is enthusiastic therein is not liable to dissipate and fritter away his time in things that are worthless and injurious to his character. Second, many principles involved in his study are of direct value in practice. Take occlusion: how important it is to understand this tremendous force in our everyday work; take tooth-forms: how important it is to know the forms of the human teeth in all their details and as to how they reach their present types; and also the functions of the teeth: how important it is to know these for the purpose of their proper restoration to functional effectiveness.

There are many other things that might be spoken of in this interesting study, but I hope enough has been said to interest you, and perhaps to stimulate investigation in some of the branches suggested this morning.—*Western Dental Journal.*

